# *LRP4* ‐Related Lethal Syndromic Form of Syndactyly in Limousin Cattle

**DOI:** 10.1002/age.70090

**Published:** 2026-03-19

**Authors:** Joana Jacinto, Jessica Gearing, Aurélien Capitan, Ana‐Maria Zorlescu, Arthur Otter, Cord Drögemüller

**Affiliations:** ^1^ Clinic for Ruminants, Vetsuisse Faculty University of Bern Bern Switzerland; ^2^ Institute of Genetics, Vetsuisse Faculty, University of Bern Bern Switzerland; ^3^ INRAE, AgroParisTech GABI, Université Paris‐Saclay Jouy‐en‐Josas France; ^4^ Animal and Plant Health Agency Shrewsbury UK

Syndactyly in cattle (OMIA:000963‐9913), also known as mulefoot, is a congenital malformation within the polydactyly–syndactyly–triphalangism category, characterized by the fusion or incomplete separation of the two fully developed digits of the bovine foot (Leipold et al. 1969; Jacinto et al. 2025). This bovine single‐gene disorder exhibits variable expression and is most commonly observed in the forelimbs. However, all four limbs can be affected following a front‐to‐rear and left‐to‐right gradient (Leipold et al. 1969; Drögemüller and Distl 2006). To date, five recessively inherited causal protein‐changing variants in the bovine *LRP4* gene have been identified in Holstein, Angus, and Simmental × Charolais crosses showing non‐syndromic forms of syndactyly (Jacinto et al. 2025; Drögemüller et al. 2007a; Johnson et al. 2006a; Duchesne et al. 2006a). This gene encodes a receptor for agrin, a large proteoglycan widely expressed at neuromuscular junctions. It is associated with human congenital myasthenic syndromes, which are characterized by impaired neuromuscular signaling (Ohno et al. 2025), as well as with rare forms of skeletal disorders affecting distal limb development, including syndactyly (Li et al. 2010; Leupin et al. 2011).

Two purebred stillborn female Limousin calves, who were paternal half‐siblings, were born in a herd of 60 cows in England, with suspected syndactyly. To investigate potential infectious aetiologies, brain samples from the second calf were tested for the presence of bovine viral diarrhea virus, *Neospora* spp., and Schmallenberg virus using PCR with negative results. Gross pathology findings revealed a phenotype resembling the bovine syndactyly in all limbs of both calves with the additional presence of brachygnathia and arthrogryposis in both (Figure S1). The pathological findings were compatible with a syndromic form of syndactyly.

Pedigree analysis revealed multiple inbreeding loops and showed that the dams of the two cases were related in the second generation. Based on the observed congenital abnormalities and the pedigree analysis, a monogenic recessively inherited form of syndromic syndactyly was suspected.

Biological material for genetic investigation was available for one trio. Therefore, short‐read whole‐genome sequencing (WGS) using genomic DNA obtained from tissue of case 1, EDTA‐blood from its dam and semen from its sire was performed as described before (Jacinto et al. 2025). The sequenced reads were mapped to the ARS‐UCD1.2 cattle reference genome (National Center for Biotechnology Information 2018), resulting in an average read depth of approximately 14.8× and single‐nucleotide variants and small indel variants were called as previously described (Jacinto et al. 2025). The genome of the trio was compared with a total of 6643 control genomes sequenced as part of successive resequencing projects in Switzerland (*n* = 1208 genomes) and France (*N* = 895) (Besnard et al. 2024; Boussaha et al. 2016, 2025), and the variant catalogue from run 9 of the 1000 Bull Genomes Project (*N* = 5116 genomes, including 576 from the Swiss Comparative Bovine Resequencing Project). Variant classification and prioritization as well as *in silico* assessment of the molecular consequences were performed as previously described (Jacinto et al. 2025).

Assuming a recessive mode of inheritance with obligate heterozygous carrier parents, the WGS‐trio approach identified seven protein‐coding variants, six of which were present in a heterozygous state in a varying number of controls from different breeds (Table S1). However, only one private variant was found to affect *LRP4*, which is a functional candidate gene (Table S1). This homozygous missense variant in exon 2 of the *LRP4* gene, which affects the low‐density lipoprotein receptor domain (Chr15:g.76826546C>A; NM_001077843.1:c.170G>T; NP_001071311.1:p.Cys57Phe; Figure S1) was classified as a candidate pathogenic variant (Boeykens et al. 2024; Richards et al. 2015) (Table S2). Additionally, the variant was predicted by MutPred (Pejaver et al. 2017) to alter the metal binding and transmembrane protein, and to lead to loss of disulfide linkage at the cystine 57 of the encoded LRP4. The variant *LRP4* allele was absent in all the controls used, including 124 purebred Limousin, indicating that it may be a rare deleterious allele present in the British Limousin population.

Despite the presence of different *LRP4* harmful alleles in different cattle breeds, the phenotypic presentation is largely consistent across all cases. (Jacinto et al. 2025; Drögemüller et al. 2007a; Johnson et al. 2006a; Duchesne et al. 2006a) In contrast, we report here the first syndromic form of *LRP4*‐related syndactyly in cattle. Interestingly, in humans missense variants in *LRP4* are associated with the recessively inherited Cenani‐Lenz syndactyly syndrome (OMIM:212780) that is characterized by severe syndactyly of hands and feet, mild facial dysmorphism and kidney anomalies (Li et al. 2010).

We report the first *LRP4*‐related lethal syndromic form of syndactyly in cattle. Our findings enhance our understanding of bovine syndactyly, highlighting the potential for both allelic and phenotypic heterogeneity associated with pathogenic *LRP4* variants. The first report of this congenial condition and apparent geographical confinement of this causal allele suggests a possible recent origin and a local founder effect. This highlights the need for targeted genetic screening to prevent its further spread within the British Limousin breeding population. It is also recommended that the occurrence of this allele be monitored in other Limousin populations.

## Author Contributions

**Joana Jacinto:** conceptualization, formal analysis, investigation, visualization, writing – original draft, writing – review and editing. **Jessica Gearing:** formal analysis, methodology, software, data curation. **Aurélien Capitan:** software, methodology, validation. **Ana‐Maria Zorlescu:** methodology, formal analysis, investigation. **Arthur Otter:** methodology, investigation, writing – review and editing, formal analysis, funding acquisition. **Cord Drögemüller:** conceptualization, funding acquisition, project administration, writing – review and editing.

## Funding

Joana Jacinto is supported in part by the Arbeitsgemeinschaft Schweizerischer Rinderzüchter (ASR), and the Swiss Federal Office for Agriculture (BLW) and by the Faculty Clinical Research Platform (FCRP) of the Vetsuisse Faculty of the University of Bern. Funding for the postmortem examination and any diagnostic testing completed at the Animal and Plant Health Agency (APHA) as part of this investigation was provided by Defra and the Welsh Government through the APHA's “Scanning surveillance for Diseases of Cattle in England and Wales” (ED1000) project.

## Conflicts of Interest

The authors declare no conflicts of interest.

## Supporting information


**Figure S1:** *LRP4* missense variant in a Limousin calf with a lethal syndromic form of syndactyly. (A) Phenotype of the affected stillborn calf (case 1). Syndactyly is present in all four limbs. The forelimbs show mild shortening with arthrogryposis, resulting in flexion of the carpal joints and digits. In the hindlimbs, all bones except the femora are markedly shortened, with the distal segments particularly affected; these are medially rotated and partially fused. Brachygnatia is also present. (B) The hindlimbs are shown in particular, displaying severe bilateral syndactyly. (C) Schematic representation of the bovine LRP4 protein. The previously reported variants associated with syndactyly are indicated with dotted lines and the newly identified p.Cys57Phe variant in the Limousin calf is indicated with a red arrow. The previously reported causal alleles include: p.Gly907Arg in Simmental‐Charolais calves (OMIA variant ID: 768) (Drögemüller et al. 2007b), p.Gly1199Ser in Simmental calves (OMIA variant ID: 769) (Drögemüller et al. 2007b), p.Arg494Cys (OMIA variant ID: 1844), and p.Asn1621_Gly1622delinsLysCys (OMIA variant ID: 627) in Holstein calves (Jacinto et al. 2025; Duchesne et al. 2006b) and a splice variant r.spl in Angus calves (OMIA variant ID: 378) (Johnson et al. 2006b). (D) Multiple sequence alignment of the LRP4 protein encompassing the region of the affected residue reveals complete evolutionary conservation across species.

**Table S1:** Detailed description of the private homozygous protein‐changing variants in the Limousin calf affected by a lethal syndromic form of syndactyly with obligate carrier parents and their frequencies in the global control cohort.

**Table S2:** Detailed description and classification of the identified *LRP4* missense variant.

## Data Availability

The WGS data (sample accession: case – ERS24610420, dam – ERS24610421 and sire – ERS24610422, project accession: PRJEB83441) are available at the European Nucleotide Archive (www.ebi.ac.uk/ena). French WGS data are also publicly available, as previously reported previously (Besnard et al. 2024; Boussaha et al. 2016, 2025).
