# Supplementary figures and images for: LRP4 ‐Related Lethal Syndromic Form of Syndactyly in Limousin Cattle
Source: Anim Genet. 2026 Mar 19;57(2):e70090. doi: 10.1002/age.70090 (PMC13001133; doi:10.1002/age.70090)

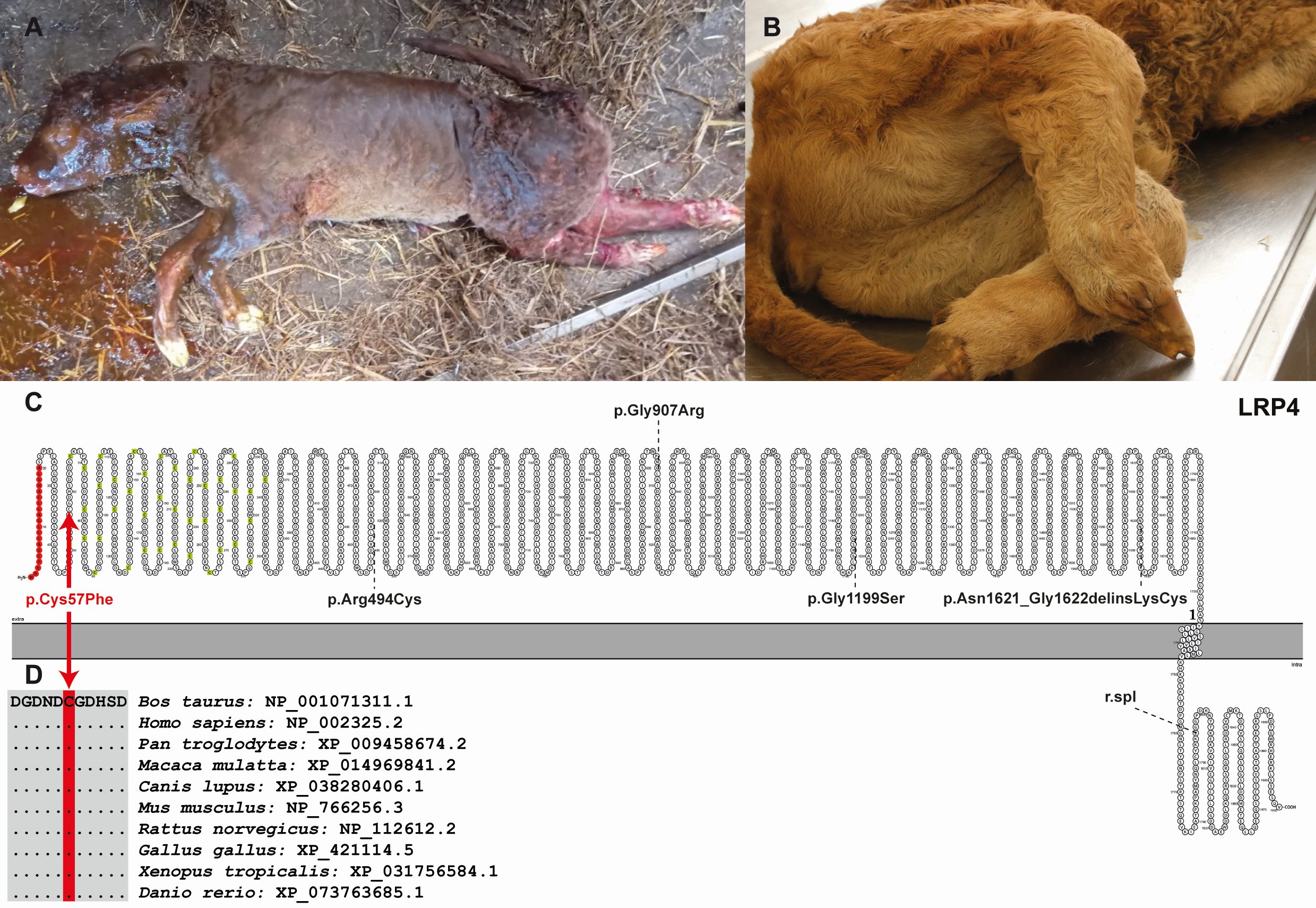

Supplement: Supplementary file 1 — Figure S1: LRP4 missense variant in a Limousin calf with a lethal syndromic form of syndactyly. (A) Phenotype of the affected stillborn calf (case 1). Syndactyly is present in all four limbs. The forelimbs show mild shortening with arthrogryposis, resulting in flexion of the carpal joints and digits. In the hindlimbs, all bones except the femora are markedly shortened, with the distal segments particularly affected; these are medially rotated and partially fused. Brachygnatia is also present. (B) The hindlimbs are shown in particular, displaying severe bilateral syndactyly. (C) Schematic representation of the bovine LRP4 protein. The previously reported variants associated with syndactyly are indicated with dotted lines and the newly identified p.Cys57Phe variant in the Limousin calf is indicated with a red arrow. The previously reported causal alleles include: p.Gly907Arg in Simmental‐Charolais calves (OMIA variant ID: 768) (Drögemüller et al. 2007b), p.Gly1199Ser in Simmental calves (OMIA variant ID: 769) (Drögemüller et al. 2007b), p.Arg494Cys (OMIA variant ID: 1844), and p.Asn1621_Gly1622delinsLysCys (OMIA variant ID: 627) in Holstein calves (Jacinto et al. 2025; Duchesne et al. 2006b) and a splice variant r.spl in Angus calves (OMIA variant ID: 378) (Johnson et al. 2006b). (D) Multiple sequence alignment of the LRP4 protein encompassing the region of the affected residue reveals complete evolutionary conservation across species. [file AGE-57-0-s003.tif]
